# Arrhythmic mitral annular disjunction or not arrhythmic mitral annular disjunction: that is the problem?

**DOI:** 10.1093/ehjcr/ytaf304

**Published:** 2025-06-27

**Authors:** Annagrazia Cecere, Martina Perazzolo Marra

**Affiliations:** Department of Cardiac, Thoracic, Vascular Sciences and Public Health, University of Padua, Via Giustiniani 2, Padova 35128, Italy; Department of Cardiac, Thoracic, Vascular Sciences and Public Health, University of Padua, Via Giustiniani 2, Padova 35128, Italy

**This editorial refers to ‘A tale of two MADs: a case series’, by K. Stankowski *et al.*  https://doi.org10.1093/ehjcr/ytaf266.**

Recently, the mitral annular disjunction (MAD) aroused great interest in the scientific community regarding its prevalence and arrhythmic role in patients with mitral valve prolapse (MVP). The role of MAD is traditionally considered as part of morphological features of MVP; however, the variability between different imaging techniques remains a limitation for large population study. First described by Bharati *et al.*^1^ in a 45-year-old woman with arrhythmic MVP died suddenly, MAD is an abnormal atrial displacement of the posterior mitral leaflet hinge point. Hutchins *et al.*,^2^ analysing a single block of atrioventricular tissue, identified this anatomical disjunction in 23/25 hearts with bileaflet MVP, speculating on the essential MAD role in the leaflet redundancy. However, after few years, Angelini *et al.*,^3^ considering the entire atrioventricular junctions in 13 hearts, recognized MAD in 12 of them. So, MAD gave the impression to be an anatomical variation of the mitral annulus, also evident in normal hearts (*Figure 1A–D*).

**Figure 1:**
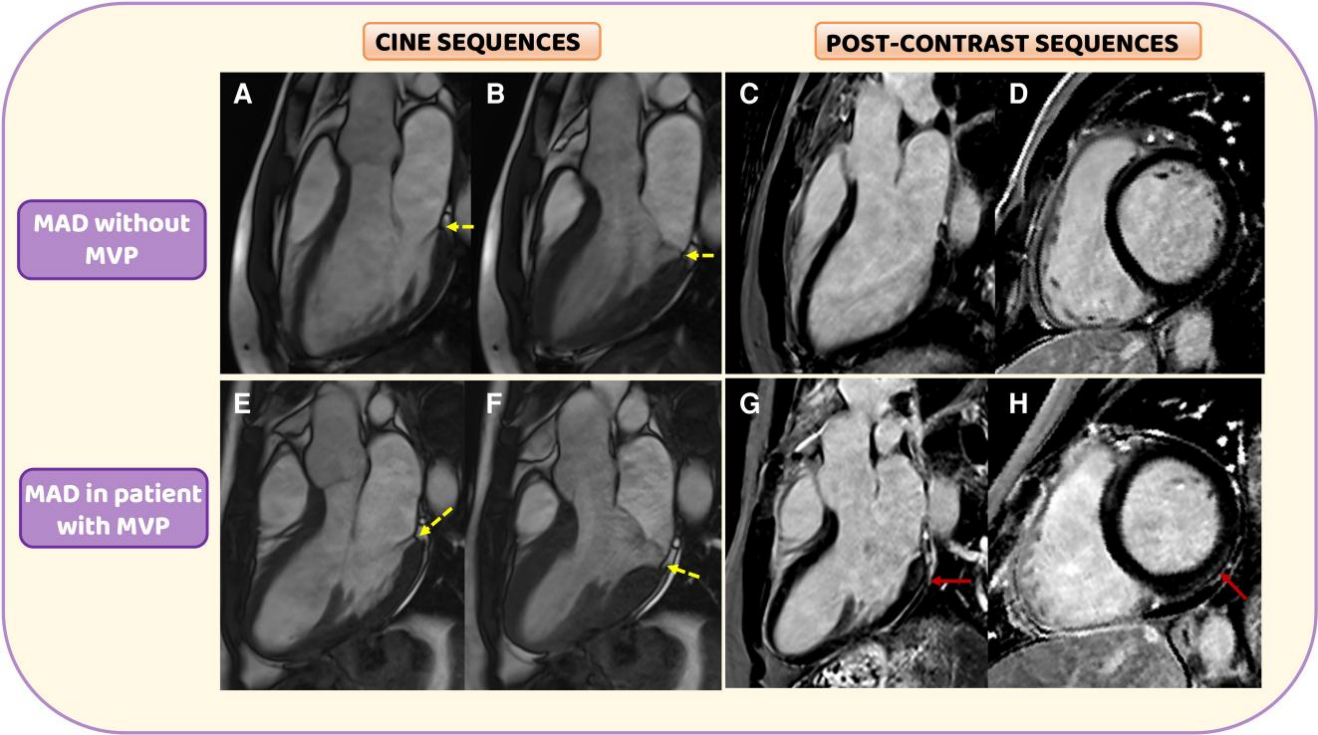
Cardiac magnetic resonance cine and post-contrast sequences in patients with mitral annular disjunction. On the superior panel (*A–D*), a representative case of a 36-year-old man with mitral annular disjunction on the cine imaging (*A* and *B*) in absence of mitral valve prolapse and myocardial fibrosis on the post-contrast sequences (*C* and *D*). On the inferior panel (*E* and *F*), a case of 28-year-old female with mitral annular disjunction and mitral valve prolapse, presenting a sub-epicardial stria of late gadolinium enhancement in the left ventricular basal inferolateral wall [red arrows (*G* and *H*) in long and short axes].

Beyond its anatomic role, the presence of MAD recently emerged as a crucial risk factor for arrhythmogenesis in MVP patients. Carmo *et al.*^4^ demonstrated that the length of MAD correlated with the occurrence of non-sustained ventricular tachycardia on Holter monitoring in MVP patients. Dejgaard *et al.*^5^ assumed that the only presence of MAD, regardless of the occurrence of MVP, was strongly associated with a greater arrhythmic risk, suggesting an independent role of the MAD in the arrhythmogenesis.

In a retrospective analysis of sudden cardiac deaths in young patients in Veneto region, MAD resulted correlated with an abnormal systolic motion of the left ventricular (LV) basal inferolateral wall, well known as *curling*, and fibrosis.^6^ So, it was hypothesized that this anatomical separation between posterior mitral leaflet and LV myocardium, acting as substrate, could promote a mechanical stress (trigger) on one side on the mitral valve, responsible for myxomatous degeneration and progressive valve incompetence, and on the other on the LV basal inferolateral wall resulting, time by time, in a replacement-type fibrosis, evaluated through late gadolinium enhancement (LGE) in cardiac magnetic resonance (CMR)^7^ (*Figure 1E–H*).

After about 10 years from the first recognition of its pro-arrhythmic role, Essayagh *et al.*^8^ demonstrated that MVP patients with MAD (186/595, 31%) presented a more severe phenotypical spectrum of mitral disease (bileaflet MVP, leaflet redundancy, and great LV volumes), suggesting a progressive myxomatous degeneration. In this study, MAD emerged as an independent risk factor for arrhythmic events, even if, intriguingly, in a 10-year follow-up, MAD was not associated to a reduced overall survival. The case series presented by Stankowski *et al.*^9^ underlined the criticisms about the arrhythmic role of MAD. They presented two different clinical scenarios of echographically diagnosed MAD in young patients. In the first case, MAD was diagnosed in a 31-year-old woman, presenting exertional syncope and palpitations, without electrocardiogram abnormalities. Echocardiography revealed normal biventricular dimensions and function. A single-leaflet non-classic MVP with a 3-mm inferolateral MAD and a trivial mitral regurgitation was also recognized. No systolic *curling* or focal/microscopic myocardial fibrosis was found in the CMR. Conversely, in the second case, MAD was identified in a 32-year-old man with an aborted sudden cardiac death due to ventricular fibrillation, occurred after a run. In this case, a 10-mm inferolateral MAD was associated to a bileaflet MVP with systolic *curling*, positive Pickelhaube sign, moderate mitral regurgitation, and left chamber’s dilation. T-wave inversion in the inferolateral leads was also observed. CMR revealed increased T1 mapping values in the LV basal inferolateral wall without focal macroscopical LGE.

Confirming the strongly debated issues on the arrhythmic role of MAD, the great value of this case series is the suggestion to shift the clinician’s attention from the particular (MAD) to the general (patient’s clinical presentation). First of all, Stankowski *et al.*^9^ confirmed the MAD diagnosis does not equivalent to the occurrence of an arrhythmic event because it could be also identified in normal hearts.^3,10^ Accordingly, not all the MAD localizations are equivalent. Zugwitz *et al.*^11^ recently confirmed that, despite MAD is a common CMR finding in general population (1990/2607 patients with MAD, 76%), the inferolateral location, strongly associated with *curling* (24/134, 17.9%), was quite rare (134 patients, 5.1%). So, in order to avoid a misinterpretation of its potential arrhythmic role, it is essential to include the MAD identification in the general patient’s clinical scenario, paying careful attention to the personal and family history, electrocardiographic features, and imaging findings. In fact, in the second case presented by Stankowski *et al.*,^9^ MAD was associated to an aborted sudden cardiac death, inferolateral T-wave inversion, systolic *curling*, mitral regurgitation, left chamber’s dilation, and microscopic fibrosis, just recognized as ‘arrhythmic red flags’.^12^ Conversely, the only diagnosis of MAD, in absence of suggestive clinical and instrumental arrhythmic features, demonstrated to have a limited value in the arrhythmic risk stratification.^8–9^ In a recent study, we evaluated the determinants of ventricular arrhythmias (VAs) in 108 arrhythmic MVP patients without valve regurgitation.^13^ A mediation analysis intriguingly revealed that only *curling* had a direct and indirect, mediated by LGE, effect on VAs. Conversely, the pro-arrhythmic role of MAD was completely mediated by myocardial fibrosis. Supporting this concept, a reduction of the mechanical LV stress with a surgical insertion of a ring/prosthesis on the mitral annulus could have an impact on the VAs burden reduction because overcomes the disjunction.^8^ However, considering the actual surgical indication in patients with severe mitral regurgitation with symptoms or LV dysfunction, the potential anti-arrhythmic effect of a MV repair in MVP patients with MAD and no significant regurgitation are just speculative.

In conclusion, the arrhythmogenic role of MAD should be carefully considered on the basis of clinical scenario and instrumental findings because it emerged as an epiphenomenon without a direct effect on arrhythmogenesis in MVP patients.^13^ Conversely, *curling*, as the expression of the abnormal LV myocardium contraction, could act as *primum movens* in the mechanical stress, demonstrated in MVP patients, and it could be directly responsible for arrhythmogenesis.^13^

Further studies will be necessary to confirm this clinical-oriented approach to the MAD identification in MVP patients, but this case series raises important steps in this direction.

## Data Availability

All data are included in the publication.

## Lead author biography

Annagrazia Cecere, MD, PhD Student, is an Assistant Professor of Cardiology at the University of Padua. Her research focuses on cardiovascular magnetic resonance, arrhythmic mitral valve prolapse, and rare arrhythmic cardiomyopathies. She has published more than 50 indexed papers (h-index 11) and delivered over 60 invited lectures. Dr Cecere holds ESC Level III Certification in Cardiovascular Magnetic Resonance and serves on the editorial boards of several indexed peer-reviewed journals. As an educator, she teaches core cardiology courses to medical students and supervises residents and post-graduate fellows during clinical and research rotations. Finally, she is a lecturer in a second-level Master's programme in cardiac magnetic resonance and rare arrhythmic cardiomyopathies at the University of Padua.

**Funding:** No funding to declare.
